# Automated Classification of Papillary Renal Cell Carcinoma and Chromophobe Renal Cell Carcinoma Based on a Small Computed Tomography Imaging Dataset Using Deep Learning

**DOI:** 10.3389/fonc.2021.746750

**Published:** 2021-11-18

**Authors:** Teng Zuo, Yanhua Zheng, Lingfeng He, Tao Chen, Bin Zheng, Song Zheng, Jinghang You, Xiaoyan Li, Rong Liu, Junjie Bai, Shuxin Si, Yingying Wang, Shuyi Zhang, Lili Wang, Jianhui Chen

**Affiliations:** ^1^ Department of Urology, Fujian Medical University Union Hospital, Fuzhou, China; ^2^ Institute for Empirical Social Science Research, Xi’an Jiaotong University, Xi’an, China; ^3^ School of Statistics and Mathematics, Central University of Finance and Economics, Beijing, China; ^4^ School of Electrical and Computer Engineering, University of Oklahoma, Norman, OK, United States; ^5^ Department of Radiology, Fujian Medical University Union Hospital, Fuzhou, China; ^6^ Department of Pathology, Fujian Medical University Union Hospital, Fuzhou, China; ^7^ School of Medicine, Fujian Medical University, Fuzhou, China; ^8^ School of Medicine, Xiamen University, Xiamen, China

**Keywords:** CNN—convolutional neural network, PRCC, papillary renal cell carcinoma, ChRCC,·chromophobe-primary renal cell carcinoma, cancer image classification

## Abstract

**Objectives:**

This study was conducted in order to design and develop a framework utilizing deep learning (DL) to differentiate papillary renal cell carcinoma (PRCC) from chromophobe renal cell carcinoma (ChRCC) using convolutional neural networks (CNNs) on a small set of computed tomography (CT) images and provide a feasible method that can be applied to light devices.

**Methods:**

Training and validation datasets were established based on radiological, clinical, and pathological data exported from the radiology, urology, and pathology departments. As the gold standard, reports were reviewed to determine the pathological subtype. Six CNN-based models were trained and validated to differentiate the two subtypes. A special test dataset generated with six new cases and four cases from The Cancer Imaging Archive (TCIA) was applied to validate the efficiency of the best model and of the manual processing by abdominal radiologists. Objective evaluation indexes [accuracy, sensitivity, specificity, receiver operating characteristic (ROC) curve, and area under the curve (AUC)] were calculated to assess model performance.

**Results:**

The CT image sequences of 70 patients were segmented and validated by two experienced abdominal radiologists. The best model achieved 96.8640% accuracy (99.3794% sensitivity and 94.0271% specificity) in the validation set and 100% (case accuracy) and 93.3333% (image accuracy) in the test set. The manual classification achieved 85% accuracy (100% sensitivity and 70% specificity) in the test set.

**Conclusions:**

This framework demonstrates that DL models could help reliably predict the subtypes of PRCC and ChRCC.

## Introduction

With the continuous advancement of imaging technology and increasing awareness of the public for early cancer screening, the detection rate of renal masses is increasing (1). In China, most renal masses are kidney cancer. The incidence of kidney cancer in the Chinese population continues to increase (2). Existing methods can meet the need to distinguish clear cell carcinoma from non-clear cell carcinoma. However, the differentiation between subtypes of non-clear carcinoma may be difficult because of the lack of a quantitative evaluation of images, especially from the early-stage cancers, which usually present atypically (3). Papillary renal cell carcinoma (PRCC) and chromophobe renal cell carcinoma (ChRCC) are the most common types of non-clear cell carcinoma and are characterized by a unique molecular morphology (4). PRCC is associated with activating germline mutations in MET (type I) and activation of the NRF2–ARE pathway (type II) (5). Typical genetic changes in ChRCC are deletions of chromosomes Y, 1, 2, 6, 10, 13, 17, and 21 (6). The differences in originating factors and driver genes between the two subtypes lead to different treatment options and prognoses (7, 8). There is some differentiation between PRCC and ChRCC in imaging findings: PRCC presents as cysts, necrosis, and calcification, while ChRCC presents as central wheel-shape enhancement (9). In low stage or small size masses, however, these characteristics mentioned above are atypical, which usually cause a difficult diagnosis. In addition, according to previous reports (10), the accuracy and sensitivity of the manual classification of PRCC/ChRCC are 61.8% and 84.5%, respectively, which cannot meet this need. Therefore, in the clinic, it is difficult to provide a highly accurate manual subtype differentiation between PRCC and ChRCC, and this remains to be a challenge.

Recently, with the rapid development of computer hardware and deep learning (DL) theory, artificial intelligence (AI) has been widely applied in radiological image processing for classification and is rapidly developing (11). Notably, the efficacy of DL-based models for the radiological diagnosis of several tumors [e.g., breast cancer (12), liver cancer (13), and lung masses (14)] is superior to that of manual processing according to previous studies (15). Convolutional neural networks (CNNs) and improved models have been widely used for medical image processing (16). DL-based oncological radiological characterization has shown value in medical fields (11, 15, 16). CNNs and their improved models are currently one of the hot spots in the field of medical image processing. Image processing based on this type of model for assisting in renal tumor examinations has achieved promising results and suggests the possibility of solving the challenges associated with the radiological differentiation of PRCC and ChRCC.

In this study, DL was utilized to classify PRCC and ChRCC from computed tomography (CT) datasets. The current study aimed to exploit DL-based models for renal cell carcinoma subtype classification based on small datasets so that the classification can be implemented in some scenarios without high-performance hardware or shortage of rare subtypes cases, to better promote the accuracy of radiological diagnosis.

## Methods

**Figure 1:**
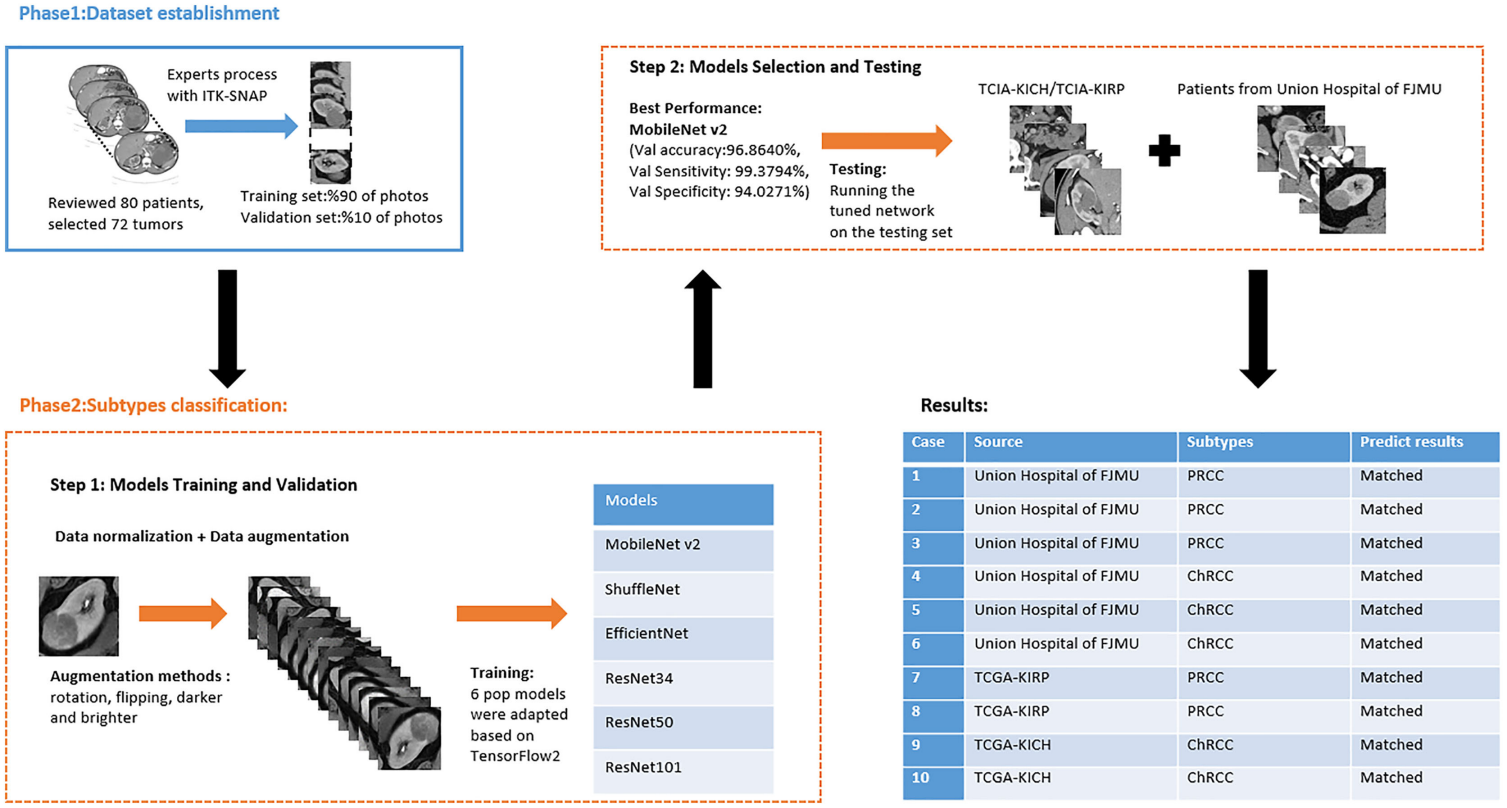
Flowchart of automated PRCC/ChRCC classification using computer vision.

Institutional review board approval was obtained. The requirement for written informed patient consent was waived. A retrospective review of PRCC and ChRCC patients at Fujian Medical University Union Hospital was performed between 2012 and 2021. Ethical approval was obtained from the Institutional Ethics Committee of Fujian Medical University Union Hospital (No. 2021WSJK033). According to the Helsinki Declaration, all patients (or their legal clients) provided written informed consent before obtaining their clinical, radiological, and pathological data. The framework used to develop an automated method for the differentiation of these two subtypes was comprised of two phases (**Figure 1**): 1) CT scan data, clinical data, and pathological data were gathered and digitized, followed by tumor lesion segmentation and labeling by experts in the radiology department (dataset establishment); and 2) training neural networks; assessing the accuracy, sensitivity, and specificity of the models; and verifying model efficiency through comparison with the pathological diagnosis of new cases (subtype classification).

### Phase 1

#### Dataset Establishment

Patients with a pathological diagnosis obtained by biopsy or surgical resection were included in this study. In addition, 80 patients with available arterial/cortical/nephrogenic phase CT image sequences were reviewed (42 with PRCC and 38 with ChRCC). After randomly selecting 6 cases (3 PRCCs and 3 ChRCCs) for testing sets, the images of 74 tumors (39 PRCCs and 35 ChRCCs) were used to build the datasets. The CT images were obtained using various radiology scanners and non-standard protocols. Arterial phase sequences were preferred when multiple phases existed. Whole sequences were retrieved and exported utilizing the hospital radiological database. The window settings were 40 HU (width) and 400 HU (level). Based on the clinical and pathological data, ROIs of sequences were segmented, labeled, and exported with ITK-SNAP by two abdominal radiologists who have experience of more than 10 years in the diagnosis of urinary system tumor. After cross-validation, images that were exported in.jpg size included 857 images of ChRCCs and 997 images of PRCCs. Labeling was applied in the non-graphical layer so that each slice filename contained the case number, gender, age, and histological subtypes. After resizing, images comprised matrices with 256 * 256 pixels in the axial planes. The dataset was divided into the training set and validation set (90% for the training set and 10% for the validation set).

### Phase 2

#### Subtype Classification

##### Model Training and Validation

**Figure 2:**
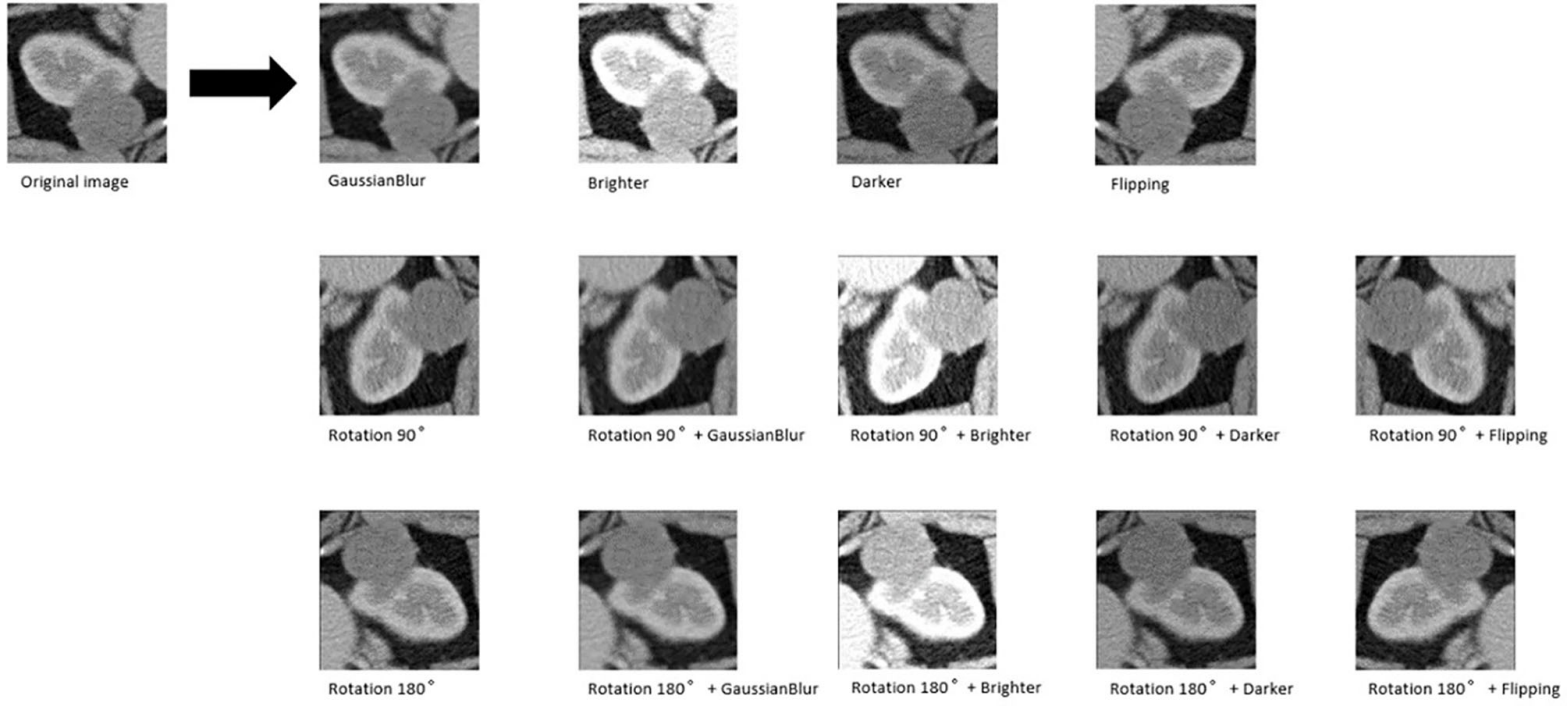
An example of data augmentation processing. Based on the geometric transformations (rotation and flipping), the Gaussian blur, brighter, and darker were applied, which finally achieved 15× amplification.

Six pop models [MobileNetV2 (17), EfficientNet (18), ShuffleNet (19), ResNet-34, ResNet-50, and ResNet-101 (20)] were adapted for dichotomy based on TensorFlow 2.4.12. Preprocessing involved normalization and augmentation (including Gaussian blur, rotation, flipping, brighter, and darker) (**Figure 2**). In addition to data augmentation, ConvBNReLU (convolution + batch normalization + ReLU) was applied to avoid overfitting. The learning rate was initially set as 0.005 and was optimized by the Adaptive Moment Estimation (ADAM) optimization algorithm in every training phase. The batch size was set as 24. For model training, a desktop workstation with an Intel^®^ Xeon^®^ E5-2678 v3 CPU and an NVIDIA GeForce RTX 2080Ti (11 GB) GPU was used. A list of model parameters, training results, and validation/accuracy results is provided in **Table 1**.

**Table 1:** The results of CNN-based networks for classification task training and validation and the testing results of the models.

Models	Parameters	Best validation accuracy	Testing results (case)
MobileNetV2	Total: 2,261,827 Trainable: 2,226,434	96.8640%	Accuracy: 100% Sensitivity: 100% Specificity: 100%
ShuffleNet	Total: 1,272,859 Trainable: 1,256,679	97.3074%	Accuracy: 83.3334% Sensitivity: 92.3077% Specificity: 72.7273%
EfficientNet	Total: 4,053,414 Trainable: 4,011,391	Cannot converge	NA
ResNet-34	Total: 21,829,058 Trainable: 21,812,034	93.6404%	Accuracy: 91.6667% Sensitivity: 84.6154% Specificity: 100%
ResNet-50	Total: 25,662,403 Trainable: 25,609,283	Cannot converge	NA
ResNet-101	Total: 44,706,755 Trainable: 44,601,411	Cannot converge	NA

NA, not available.

##### Model Selection and Testing

Based on the results of the training step, MobileNetV2, ShuffleNet, and ResNet-34 were selected as the testing models. A special test set of PRCC/ChRCC samples was established in two parts (**Table 2**): 1) reviewing the new cases in 2021, including six patients (three with PRCC, three with ChRCC); and 2) reviewing cases in The Cancer Imaging Archive (TCIA) datasets, including four patients (two with PRCC from the TCGA-KIRP dataset, two with ChRCC from the TCGA-KICH dataset). Slices were processed by abdominal radiologists, and for each case, three photographs were selected randomly. To assess efficiency from different views, two accuracy values were calculated. 1) Case accuracy: if correctly identified photographs were >2, this case was regarded as correctly identified. Case accuracy was used to reflect the percentage of correct cases. 2) Sample accuracy: this was used to show the proportion of correct images among all images. The accuracy, sensitivity, and specificity of these models were computed. In order to show the efficiency of manual processing, two radiologists were invited to distinguish these cases. Objective measure indexes of manual prediction were also calculated.

**Table 2:** Information of test sets, comparison result of automated model prediction, and the result of model performance in the validation dataset.

Case	Source	Subtypes	Gender	Age	Sample	Automated prediction	Manual prediction
1	Union Hospital of FJMU	PRCC	Female	60	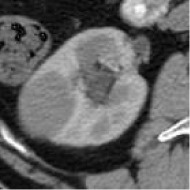	Matched	1. Matched 2. Matched
2	Union Hospital of FJMU	PRCC	Male	58	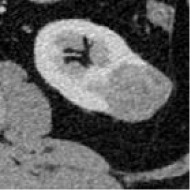	Matched	1. Matched 2. Matched
3	Union Hospital of FJMU	PRCC	Male	57	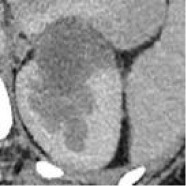	Matched	1. Matched 2. Matched
4	Union Hospital of FJMU	ChRCC	Male	62	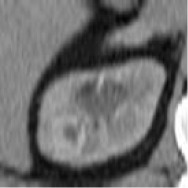	Matched	1. Matched 2. Matched
5	Union Hospital of FJMU	ChRCC	Female	41	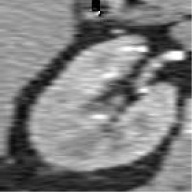	Matched	1. Matched 2. Mismatched
6	Union Hospital of FJMU	ChRCC	Female	62	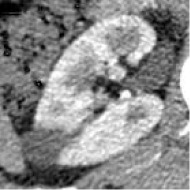	Matched	1. Matched 2. Matched
7	TCGA-KIRP	PRCC	–	–	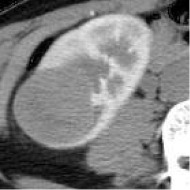	Matched	1. Matched 2. Matched
8	TCGA-KIRP	PRCC	–	–	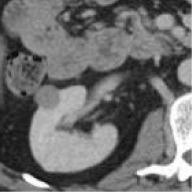	Matched	1. Matched 2. Matched
9	TCGA-KICH	ChRCC	–	–	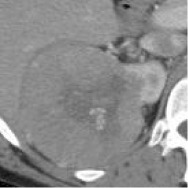	Matched	1. Matched 2. Mismatched
10	TCGA-KICH	ChRCC	–	–	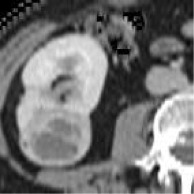	Matched	1. Mismatched 2. Matched
Validation accuracy		96.8640%					
Validation sensitivity		99.3794%					
Validation specificity		94.0271%					
Test accuracy (case)		100%					
Test sensitivity (case)		100%					
Test specificity (case)		100%					
Test accuracy (image)		93.3333%					
Test sensitivity (image)		88.2353%					
Test specificity (image)		86.6667%					
Manual accuracy		85% (90% and 80%)					
Manual sensitivity		100%					
Manual specificity		70% (80% and 60%)					

## Results

**Figure 3:**
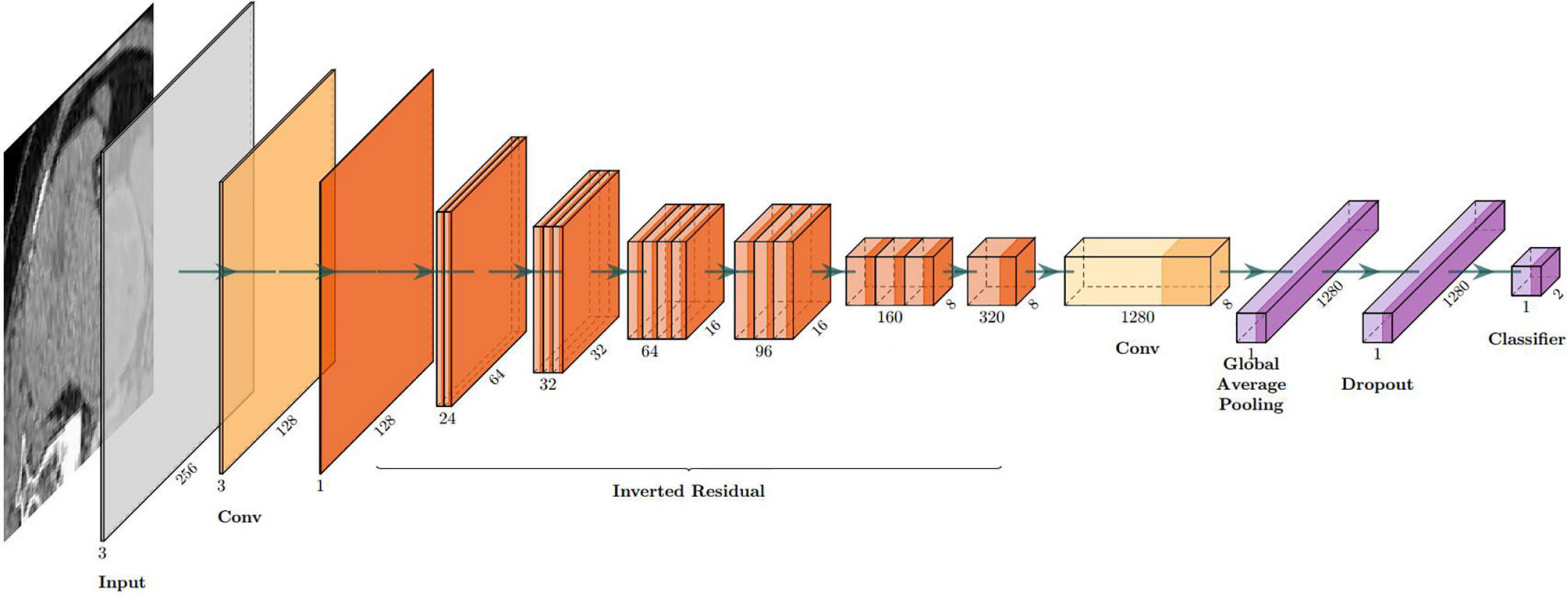
The visual structure of MobileNetV2.

**Figure 4:**
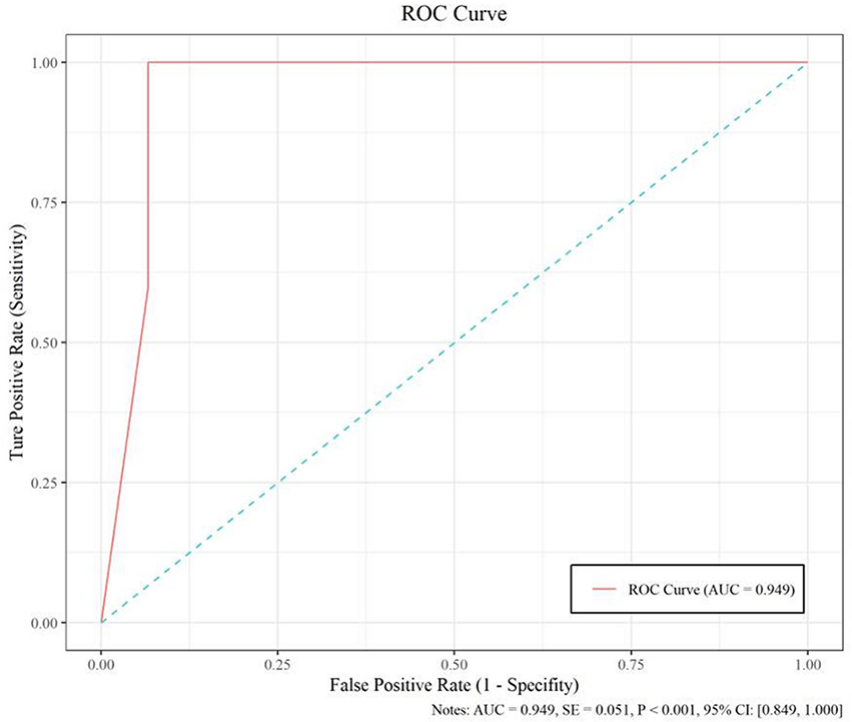
The performance measure of this model, including the ROC curve, AUC (0.949), and confidence intervals (0.849, 1.000).

The model based on MobileNetV2 (**Table 3** and **Figure 3**) performed best for tumor subtype diagnosis. The automated method achieved 96.8640% accuracy in the validation dataset (99.3794% sensitivity, 94.0271% specificity). Due to all correctly matching, case accuracy, case sensitivity, and case specificity were all achieved 100%. For every single photograph, image accuracy achieved 93.3333% in the testing dataset (88.2353% sensitivity and 86.6667% specificity). The AUC was 0.9489, and the *p*-value was less than 0.001. Resource occupancy was less while training and predicting (less than 10 GB of accelerated graphics memory occupied), which means that this model can be applied to low-performance hardware. The manual method achieved 85% accuracy (100% sensitivity, 70% specificity) in the testing dataset. The results are provided in **Table 2** and **Figure 4**.

**Table 3:** The structure of MobileNetV2.

Layer (functions)	Output shape	Stride	Filter shape
Input layer	None, 256, 256, 3	/	/
Conv1 (Conv+BN+ReLU6)	None, 128, 128, 32	2	3 * 3 * 32
Inverted_residual (linear)	None, 128, 128, 16	1	1 * 1 * 32 * 16
Inverted_residual_1 (ReLU6)	None, 64, 64, 24	2	3 * 3 * 16 * 24
Inverted_residual_2 (linear)	None, 64, 64, 24	1	1 * 1 * 24
Inverted_residual_3 (ReLU6)	None, 32, 32, 32	2	3 * 3 * 24 * 32
Inverted_residual_4 (linear)	None, 32, 32, 32	1	1 * 1 * 32
Inverted_residual_5 (linear)	None, 32, 32, 32	1	1 * 1 * 32
Inverted_residual_6 (ReLU6)	None, 16, 16, 64	2	3 * 3 * 32 * 64
Inverted_residual_7 (linear)	None, 16, 16, 64	1	1 * 1 * 64
Inverted_residual_8 (linear)	None, 16, 16, 64	1	1 * 1 * 64
Inverted_residual_9 (linear)	None, 16, 16, 64	1	1 * 1 * 64
Inverted_residual_10 (linear)	None, 16, 16, 96	1	1 * 1 * 64 * 96
Inverted_residual_11 (linear)	None, 16, 16, 96	1	1 * 1 * 96
Inverted_residual_12 (linear)	None, 16, 16, 96	1	1 * 1 * 96
Inverted_residual_13 (ReLU6)	None, 8, 8, 160	2	3 * 3 * 96 * 160
Inverted_residual_14 (linear)	None, 8, 8, 160	1	1 * 1 * 160
Inverted_residual_15 (linear)	None, 8, 8, 160	1	1 * 1 * 160
Inverted_residual_16 (linear)	None, 8, 8, 320	1	1 * 1 * 160 * 320
Conv (ReLU6)	None, 8, 8, 1,280	1	1 * 1 * 320 * 1,280
Global average pooling	None, 1,280	1	Pool 8 * 8
Dropout	None, 1,280	1	Probability = 0.2
Classifier (ReLU)	None, 2	/	Classifier

## Discussion

Before a clinical treatment strategy is developed, the gold standard for the differentiation of subtypes is pathological diagnosis by histological biopsy. Nevertheless, this invasive operation may increase the possibility of needle tract implantation and the metastasis of malignant tumors, as well as the risks of bleeding, infection, and damage to surrounding organs caused by puncture operations. Furthermore, the missed diagnosis rate is approximately 30% (21). An ideal renal tumor diagnosis method should avoid unnecessary damage to patients and potential risks as much as possible while ensuring high accuracy and a high detection rate, which points to the need to further improve auxiliary examination image processing technology to increase sensitivity and accuracy, as it has great prospects.

The accuracy and sensitivity of a manual imaging diagnosis cannot meet current clinical diagnosis and treatment needs. In addition, there is still a lack of clinical and radiological features that can accurately predict histology. The current imaging diagnostic method has significant limitations. The accuracy and sensitivity of the manual classification of PRCC/ChRCC according to existing reports are 61.8% and 84.5% (10), which are significantly lower than those of our model. The average accuracy, sensitivity, and specificity of manual classification by our radiologists of these subtypes are 85% (90% and 80%), 100%, and 70% (80% and 60%), which are also lower than those of our model. Our MobileNet-based model also showed better efficacy than manual processing. This result provides an automated approach to the dilemma of diagnosing subtypes with radiological data and may affect the selection of surgical methods and clinical decisions.

As a typical DL algorithm, due to their interlayer parameter sharing characteristics and sparse connection characteristics of the model architecture, CNNs can realize the automated extraction of pixel-level image features without the need to establish and engineer large-scale features in advance, and due to the real-time nature of the model itself, features such as flexibility, associative information storage, and backpropagation algorithm change weights can achieve higher processing accuracy with manual data than traditional machine learning, prompting high-throughput automation based on the feasibility of CNN/DCNN models for imaging and omics analyses. The application of comprehensive digitized clinical data, radiological images, and pathological data has paved the way for automated processing methods based on AI for radiological data processing in the future. In recent years, various studies have started utilizing complete digital radiological and clinical data for segmentation and classification (22, 23), verifying the feasibility of this scheme. In nephrology oncology, study interests that incorporated AI started focusing on subtype classification. Tanaka et al. (24), based on the Inception-v3 CNN model and MR images, identified benign and malignant renal masses (≤4 cm) on images with an accuracy rate of 88%. Based on a CNN model and CT images, Baghdadi et al. (25) identified benign renal oncocytoma and ChRCC on images with an accuracy rate of 95%. Zhou et al. applied transfer learning to classify benign and malignant kidney tumors with CT datasets, and the accuracy of the difference was reported to be 0.95 (26). Lee et al. developed a model that combined DL and manual feature machine learning to classify specific kidney tumor types, and the accuracy was 0.77 (27). These studies prove that the imaging differentiation of kidney tumors based on DL and dichotomy is feasible but lacks utility and requires high-performance hardware, limiting the research results to the clinic. The present models show the possibility of using a high-confidence DL-based diagnostic method for the radiological classification of PRCC/ChRCC and provide a feasible low-performance hardware program with high accuracy for different medical devices that can be applied even to a gaming laptop or a mobile workstation.

There were some new findings obtained during training and validation that have not been reported in research in the same field. First, according to the experimental results, we speculate that the valuable features of PRCC/ChRCC on CT images are commonly overlooked, which indicates that the fewer trainable parameters the model has, the better the accuracy it achieves. Although the feature capacity of the models is correlated linearly with the number of parameters, the number of parameters is seemingly correlated with fitting situations in a parabola. In the lightest model, ShuffleNet, performance is the worst in these coverage models. The best performance is from the MobileNetV2, with a bigger capacity of parameters than ShuffleNet. However, as the number of parameters is continuously increasing, the accuracy is decreasing (ResNet-34). Importantly, too many trainable parameters in this task can cause model under-/overfitting (ResNet-50/ResNet-101). The relation between accuracy and parameters during the classification of small datasets needs further explanation and selection. However, this interesting finding shows the importance of feature capacity assessment of datasets and the right choice of models with a suitable size before promoting performances. Finally, we noticed that extreme data augmentation has little effect on training. We tried several ways to augment and amplify datasets to increase their size, which obviously did not affect the accuracy of the validation dataset.

**Figure 5:**
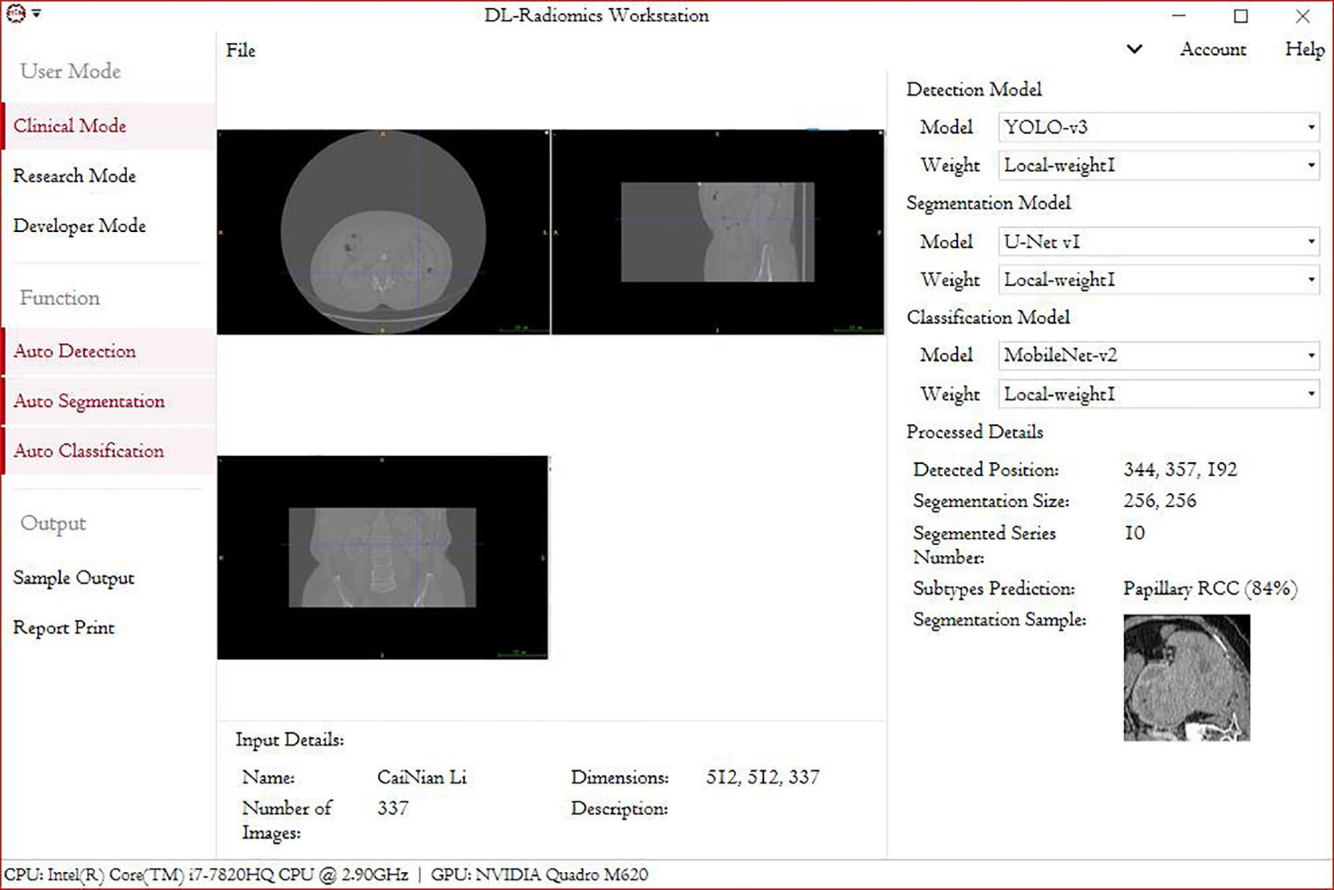
A demo of a DL-based radiomics workstation (next study).

This study had two main aims: 1) to make the automated classification methods easy to use with broad applicability to provide a highly accurate method that can be used in basic health units and deployed in medical centers with low-performance hardware and 2) to combine these methods with those used in federal studies, which can be used for multicenter studies and to increase model accuracy without the need to gather all the data. The deployment of such a model in devices at health centers will promote clinical treatment. Our future goal is to migrate this processing paradigm to other RCC subtypes. Although this study provides the first automated method for the radiological classification of PRCC/ChRCC subtypes, there are still some limitations. 1) The main limitation is the lack of multicenter validation, and the other limitations include the sizes of the training, validation, and testing datasets, which will be considered in future studies. Our methods used to avoid overfitting included data augmentation and ConvBNReLU but should have been more diverse. Also, due to the limited dataset capacity, the ROC curve was unsmoothed. 2) The underutilization of digital clinical data is another limitation. The conjunctive use of clinical and radiological data can further improve the prediction accuracy. 3) The underuse of multiphase sequences could be considered another limitation of our study. In our study, we exported images based on one patient–one phase. 4) Our dataset was mainly obtained from East Asian patients, and since a population-based analysis reported that racial disparities exist between black and white people in kidney cancer (28), the upshot of our study would have bias in the East Asian population. Multiple factors including race, gender, and age could be taken into consideration for further exploration. 5) Our validation dataset was based on a dataset from our hospital, but it was not the best choice and had a certain effect on the results of training. The ideal validation dataset should be based on three or more datasets from different institutes. The small size of the testing dataset could also have led to a controversy about the results of MobileNetV2, which need to be further tested in multiple centers. 6) Processing of datasets by experts may not be regarded as the best method. Ideally, an automated segmentation procedure contained in the pipeline may be a better choice. However, there were some barriers laid that could hardly be bypassed. We tried two proposals: individual tumor output and area output. We applied U-Net-based methods to draw ROIs and found that the methods of existing reports (29–31) could not fit the need. In studies, tumor Dice scores were lower than 0.85 generally, which meant that some parts of tumor pixels could not be contained in the processed images and some radiological characters were lost unavoidably. The reason why we finally did not use this method was that this automated segmentation/classification-combined model had a performance lower than expected. Unless the method to improve the tumor Dice score obviously is developed, the automated segmentation–classification model efficiency has a rare possibility to reach the baseline of clinical application. We also tried YOLO-v3-based detection and area segmentation; however, it did not show better performances compared with existing ML-based methods, which finally led to its abandonment. Besides the technological challenges, the main reason why we did not introduce an automated segmentation into the pipeline was that in this study we focused more on the classification, and the key point was realizing the classification of subtypes with low capacity under a smaller feature engineering preprocessing and more automated processing compared with ML-based classification methods. As a challenge in DL-based radiomics, automated segmentation is our next study orientation. We are developing a possible method to realize our proposed DL-based radiological processing series, and we are also trying to integrate several models into a DL-based radiomics workstation (**Figure 5**).

Overall, although there may be limitations in this study such as a small dataset and differences in races as well as in imaging single-center protocol, the research results may be biased to some extent. However, based on the result that a CNN-assisted diagnosis model with high diagnostic accuracy was developed in a single center of this study, it suggests that the AI research and development model adopted in this study has high clinical application potential in improving the accuracy of differential diagnosis of PRCC and ChRCC, at least in a single regional center. In the future, although there will be some difficult challenges in developing AI high diagnostic accuracy which was caused by some objective factors such as subtle potential differences in image feature led by the discrepancy between race and region and inability in high homogeneity in the imaging method, we still expect that the AI auxiliary renal tumor imaging diagnostic research can expand into different regions, different centers, and different races, together with bigger sample data to validate our conclusion, and can accurately classify as well as precisely and automatically segment multiple pathological types of renal tumor, with the aim of making it an auxiliary diagnostic imaging tool with wide clinical application prospects.

## Conclusions

To the best of our knowledge, this study provides the first automated framework for differentiating PRCC and ChRCC that could produce reliable results. This approach may be useful in improving the radiological diagnostic accuracy of RCC and, thus, benefit patients.

## Data Availability Statement

The original contributions presented in the study are included in the article/supplementary material. Further inquiries can be directed to the corresponding authors.

## Ethics Statement

Written informed consent was obtained from the individual(s) for the publication of any potentially identifiable images or data included in this article.

## Author Contributions

TZ and YZ: conceptualization, project administration, writing—original draft, and writing—review and editing. LH and TC: data curation, formal analysis, software, and visualization. BZ, JY, XL, RL, JB, SS, YW, SYZ, and SZ: investigation, methodology, and writing—review and editing. LW and JC: conceptualization, data curation, funding acquisition, investigation, project administration, supervision, validation, writing—original draft, and writing—review and editing. All authors contributed to the article and approved the submitted version.

## Funding

This work was supported by grants from the Natural Science Foundation of Fujian Province (2019J01153) and the Startup Fund for Scientific Research, Fujian Medical University (2019QH1053).

## Conflict of Interest

The authors declare that the research was conducted in the absence of any commercial or financial relationships that could be construed as a potential conflict of interest.

## Publisher’s Note

All claims expressed in this article are solely those of the authors and do not necessarily represent those of their affiliated organizations, or those of the publisher, the editors and the reviewers. Any product that may be evaluated in this article, or claim that may be made by its manufacturer, is not guaranteed or endorsed by the publisher.
